# Richmond Academy of Medicine and Surgery

**Published:** 1890-10

**Authors:** J. W. Henson

**Affiliations:** Reporter


### RICHMOND ACADEMY OF MEDICINE AND SURGERY.

[J. W. Henson, M. D., Reporter.]

August 12th, 1890.—Dr. W. W. Parker, President, in the Chair.

The President, Dr. Parker, read a

### REPORT OF A POST MORTEM IN A CASE OF OSTEO-SARCOMA.

On the first of January last he saw Mrs. C., aged 24 years, (mother of one child of 3 years and now 4 months pregnant), who had been suffering about two years with a tumor in the right groin. It was nearly the size of the fist, hard and painful on pressure. It appeared (small in size) soon after the birth of her child, and was thought by the doctor then in attendance to be an enlarged gland. Not much attention was paid to it then, as it was not very painful. It grew very gradually, and after two or three months she consulted a surgeon who pronounced it "cancer." This diagnosis shocked her so that she at once took to her bed and commenced taking opium. She remained in bed three months in the greatest distress. She finally took heart and got up and attended to her household duties. Several doctors saw her. The tumor was aspirated, and nothing but blood escaped. Electricity was used on the tumor for some time, and seemed to give great relief, but did not reduce its size. She still continued to take opium.

When Dr. Parker first saw her, she was cheerful, and quite active going about the house, and often on the street, though much emaciated. The tumor had been for some time at somewhat of a standstill and continued so a good while after he began attendance. The right leg (the right side being the one affected) was not larger than her arm in health—indeed, not so large. The muscles were shrunken and flabby, yet she walked quite well and fast—with a slight limp. The pain was intense down this leg to the heel, sometimes very severe in the calf. She had never been leeched or blistered. The leeching was tried by Dr. P. but no good was done; but small blisters, often repeated, gave great relief. There was a distinct aneurismal thrill in the tumor, which *now* filled the iliac fossa, and was as large as the doctor's fist, but oblong in shape, the smaller end downwards. The thrill proceeded from an artery running obliquely across the tumor outward.

Dr. Parker (it was now about two months since he first saw the woman) called in Dr. Isaiah H. White to see if this artery might not be tied, thinking it might be feeding the tumor. He thought that at least an exploratory incision would be justifiable, provided the lady passed safely through her confinement. She had now been pregnant six months. She was safely delivered at term of a small but healthy infant—chloroform, whiskey and ergot being used during the labor. The child was now living and growing finely.

Six weeks after her confinement, she rode out (against Dr. P.'s advice), and came home in great pain, and went to bed. So great was the pain that it was necessary to administer chloroform, as opium would not give relief. She took immense quantities of the former. He forgot to say that about three weeks after her confinement the pain *entirely* disappeared (strange to say). *She* then stopped the opium and chloroform and had great hopes of complete recovery.

Her appetite, too, became enormous. She ate six hearty meals a day, and would awake at daybreak and demand her breakfast. Her appetite reminded him of some convalescents from typhoid fever. It was surprising to see how rapidly her strength and flesh increased. Her spirits rose to the highest pitch. She was the happiest woman the doctor ever saw. No opiate of any sort was now taken.

But in exactly fifteen days the pain returned with great violence, as stated above in connection with the drive after her confinement.

Dr. White again saw her with Dr. Parker with a view to exploring the tumor. They found it had rapidly increased in size since her confinement—had extended downward, the arterial thrill was now more distinct and other adjacent vessels were enlarged.

After taking into account the uncertainty of the diagnosis, and that it would be necessary to enter the peritoneal cavity in order to thoroughly explore the tumor, both doctors concluded it would not be wise to attempt an operation. The pain increased to such an extent that she sometimes had to take two drachms of McMunn's elixir of opium every hour.

From the time she rode out until her death (July 27th), she took, in addition to immense quantities of opium, *nineteen pounds* of chloroform. Much of this was wasted, as she often took it herself. Her death was sudden. Upon rising to vomit, she fell upon her pillow and expired without a struggle.

Thirty-six hours after death, in company with Dr. White and Dr. J. Michaux, who had at one time attended her, Dr. Parker laid open the tumor by making two incisions—one over the entire length of the growth downwards and inwards; and a second (small one) across the first, and running inwards. The muscles covering the tumor looked healthy. Upon reaching the mass, he found it impossible, on passing his hand inwards, to dislodge it from the brim of the pelvis. It was bound down to the brim as far as the pubis by a dense fibrous tissue, thickened periosteum perhaps. He then withdrew his hand, and passed it outward and backward along the crest of the ilium, coming in contact with an immense amount of diseased bone, which extended backward nearly to the sacrum. In some places he could push his finger through the bone, which was honey-combed. He made an incision into the tumor, and found part of it soft—not exactly medullary, but not at all scirrhous—and of the color of pale muscular tissue.

As the origin of the disease was now plain, he did not remove the whole tumor. It evidently began in the bone or periosteum. The exciting cause may have been the lady's striking her right groin against the edge of a table about four or five years before the trouble began to manifest itself. The accident produced great pain, which lasted for some time.

Dr. Parker said that he read quite a lengthy paper before the Medical Society of Virginia two years ago against the habit of some excellent surgeons who think it their duty to be frank with these patients, and in this paper he took the ground that their course was always unwise in the case of cancer.

This patient was a beautiful woman, full of life and energy, and lost one or two years of comparative happiness by the announcement of the surgeon that the growth was a cancer.

One gratifying result of this post mortem was that it relieved the minds of the husband and mother of the idea that the patient had been neglected or the case mismanaged. It is doubtful if any operation at any time would have been beneficial in its results.

Dr. Michaux had attended this woman in her first confinement, and had her in charge when the tumor first developed. Two and a half years ago, she came to him complaining of pains in the right iliac region before the appearance of the growth. He examined her per vaginam, and by means of bi-manual palpation, suspecting some ovarian trouble, but found no enlargement of that organ or other evidences of disease.

The pains from the beginning came on in paroxysms, which, as time and the case advanced, grew more violent, and the intervals became shorter. Then a small nodule appeared in the right iliac region—growing, but not rapidly. The *length* of the pains increased too. Thinking to learn more, he used the exploring needle. He found a very dense tumor—a few drops of blood (nothing else) escaping. He declined to operate, thinking the growth malignant. Being invited by Dr. Parker, he attended the post mortem, and was still more inclined to think it malignant.

EARLY REMOVAL OF SUSPICIOUS GROWTHS ADVOCATED.

Dr. J. N. Upshur thought Dr. Parker's case a very interesting one. He had read in some journal a report in which the writer advocated the removal of suspicious growths before the appearance of any cachexia or involvement of lymphatic glands. He would like to know the result of a microscopic examination in Dr. P.'s case. The symptoms pointed to malignancy, and the character of the growth and its involvement of bone suggested the colloid variety. He didn't agree with Dr. P. in not telling the patient frankly the prognosis. He thought there was a great deal in the manner of telling. Break it to the patient so the shock and horror of it all would be taken off. He had never regretted being frank.

ADENOMA OF THE MAMMARY GLAND.

Dr. Upshur was reminded (by Dr. Michaux's remarks) of the case of a lady whom he had delivered in September last. She had a good labor and a satisfactory "getting up"—except that she had a slight continued fever during the last two weeks of her confinement. She also suffered from sore nipples, for which he had used lead nipple shields. At the seventh week he was called again to see the patient, who was suffering from supposed mammary abscess due, her mother thought, to bruising from the nipple shields. The doctor thought this impossible, as the metal was too soft. After poulticing, he gave chloroform. Fluctuaion being as distinct as he had ever felt, he lanced the breast. To his surprise only a little sanguinolent matter was discharged, and the tissue cracked under the knife like a potato. The absence of pus and the peculiar feel on cutting were both very striking features. The pain and throbbing subsided, and the part not discharging, the poulticing was stopped after a while. The pain and throbbing recommencing, the doctor went through the same process of poulticing and lancing with the same experience, except there was this time some pus. Later still he repeated the above treatment, and this time, upon continuing the poulticing, the place discharged more pus than it had done at all. He stated to the patient's mother that this was not ordinary mammary abscess, but an adenoma, being benign at the time, but that it might in the future degenerate so as to need surgical interference.

After this, under the influence of tonics, the patient's general health improved. He used potassium iodide and fluid extract gentian internally, and five per cent. oleate of mercury locally; but not succeeding in reducing the tumor, after fair trial, the treatment was discontinued and the patient urgently advised not to rub or handle the gland. She was now put on the syrup of lactophosphate of lime with very decided improvement in her general health. The baby had not been weaned from this breast because of the free flow of milk from the inferior part of the gland, and the fear of abscess in that location; but so soon as the milk could be dried up, the weaning from this breast was done. When the patient passed from under Dr. Upshur's care, she was looking as well as he had ever seen her.

She had subsequently an attack of *la grippe*, and a few weeks later cholera-morbus.

The said patient is now in Dr. Hugh M. Taylor's hands, and he (Dr. Upshur) thought the subsequent history of the case would be of interest to the Academy. He had erred (said the doctor) in two points:

*First.* He believed that if he had stated frankly the probable outcome of the trouble and had advised excision, the woman's life would have been saved or prolonged.

*Secondly.* In the light of the subsequent history, he believed he erred in lancing the breast, as no good had resulted.

This was a case which illustrated the importance of frankness and early excision. He thought the latter very important in the case of suspicious growths.

### SCIRRHUS OF THE MAMMARY GLAND.

Dr. Upshur also referred to an old lady of sixty. She had in one mammary gland an irregular hard lump, but her former physician (now dead) advised her to let it alone, because at that time it gave her no trouble.

Sometime ago she sent for Dr. Upshur, and showed him the lump. She complained of trouble in using her right arm, and tenderness of the breast. There were darting pains and *retracted nipple.* The lump was hard and irregular, though not adherent to the chest wall, and presented symptoms of scirrhus. He advised removal. He could not convince her and her friends of the nature of the trouble. He told her frankly if she waited too long it would be to late. (Caution urged in lancing the mammary gland.)

Dr. Parker thought Dr. Upshur wrong in lancing in the first case he reported. This should never be done unless there was absolutely distinct fluctuation. He was suspicious of breast tumors anyway unless he could be absolutely sure of abscess. He thought Dr. Upshur also wrong in telling the patient the diagnosis in the case of malignancy. He had as lief shoot a woman as to tell her she was cancerous. Tell her friends always, but not the patient. Thousands and thousands were made miserable by this practice. He was sustained, said the doctor, by Mr. Locke.

Dr. Upshur stated that the fluctuation was positive and unmistakable. He believed he would be in error to state that fluctuation and the peculiar feeling in cutting, before described, when taken together were characteristic of malignancy.

Dr. Hugh M. Taylor said the history of the first case reported by Dr. Upshur had been given him just about as the doctor had related it. He refused to say anything about the case, as the patient and friends, being very sensitive, preferred that it should not be spoken of.

THE DIAGNOSIS OF SCIRRHUS OF THE RECTUM IN A CHILD OF THIRTEEN YEARS CONFIRMED.

Dr. Michaux referred to the case of scirrhus of the rectum in a child of thirteen years, reported by him at the meeting of this body July 8th. Since that report he called Dr. J. S. D. Cullen in consultation, who confirmed his (Dr. Michaux's) diagnosis. The growth finally closed up the caliber of the bowel, but the family would not submit to artificial relief. The child died, the doctor thought, more from lack of drainage through the bowels than from sheer malignancy. He had only one action for fourteen days. That was just before his death. It was small in quantity, consisting of a little mucus and blood, with a very small amount of fluid fæcal matter. He thought there could now be no doubt as to the malignancy of the trouble.

POINT OF SELECTION FOR MAKING ARTIFICIAL ANUS.

Dr. M. D. Hoge, Jr., had read Dr. Hunter McGuire's report on twenty-one cases of supra-pubic cystotomy, in which Dr. McGuire said, that if he had a case upon which to operate for making an artificial anus, he would select somewhere in one of the recti muscles, near the median line, because he had noticed that the contraction of these muscles tended to close any opening that might be about the location just mentioned. This case of Dr. Michaux would have been a good one for such an operation. The boy's life might have been prolonged if such a step could have been taken after the bowel was closed.

OSTEO-SARCOMA OF THE ORBIT.

Dr. Chas. M. Shields, three months ago, saw a child eighteen months old suffering from protopsis of the right eye-ball. It began as a drooping of the upper lid. Ptosis soon gave place to protrusion of the ball. At the time he first saw it (five weeks after its commencement), the right eye-ball was about three-quarters of an inch in advance of its fellow. The cause seemed to be some growth or enlargement situated above the upper and outer part of the organ. The said growth or enlargement continued to increase in an upward and outward direction for a period of three weeks, when the doctor advised an operation. The original trouble seeming, as above stated, to be at the upper and outer part of the eye, made him suspect a cancerous enlargement of the lachrymal gland.

He went with an assistant to the patient's home, intending to put it under the influence of chloroform and examine the part, also to draw some fluid from the suspected gland for microscopic examination. He found the child, however, had a fever, and he turned the patient over to the family physician.

In a few days the said physician called in Dr. Shields, who then found the child had decided meningitis, with high fever and marked opisthotonos. There was also purpura hemorrhagica over the whole surface of the body. The child died.

The day before death there was a hemorrhage from the nose, which the doctor attributed to extension of the growth into the ethmoid bone.

A post mortem was held. A tumor had begun from the roof at the upper and outer part of the orbit. This fact, and its growing upward and outward, had originated the idea of cancer of the lachrymal gland. The growth extended over into the ethmoid, and involved it considerably. He had sent a specimen to the Army and Navy Bureau for microscopical examination. He had gotten no report. He thought the growth an osteo-sarcoma. The most peculiar feature about the whole affair was the purpura hemorrhagica over the whole surface of the body.

